# Supplementary material for: Evaluating the influential factors for life preserver donning tests
Source: PLoS One. 2021 Feb 8;16(2):e0246705. doi: 10.1371/journal.pone.0246705 (PMC7870007; doi:10.1371/journal.pone.0246705)
Supplement: S1 Table — (DOCX) [file pone.0246705.s001.docx]

S1 Table. The post hoc multiple comparisons of one-way ANOVA by LSD test.

| Donning performance | Age group | Age group | Mean  differences | Standard  error | Sig. | 95% confidence intervals | |
| --- | --- | --- | --- | --- | --- | --- | --- |
|  |  |  |  |  |  | Lower | Upper |
| Retrieving time | <20 | 20-29 | 0.950 | 0.907 | 0.297 | -0.844 | 2.744 |
|  |  | 30-39 | 1.250 | 1.954 | 0.523 | -2.612 | 5.112 |
|  |  | 40-49 | -0.310 | 0.971 | 0.750 | -2.230 | 1.610 |
|  |  | 50-59 | 0.777 | 1.363 | 0.569 | -1.917 | 3.471 |
|  | 20-29 | <20 | -0.950 | 0.907 | 0.297 | -2.744 | 0.844 |
|  |  | 30-39 | 0.300 | 2.048 | 0.884 | -3.747 | 4.347 |
|  |  | 40-49 | -1.260 | 1.148 | 0.274 | -3.529 | 1.009 |
|  |  | 50-59 | -0.173 | 1.494 | 0.908 | -3.126 | 2.780 |
|  | 30-39 | <20 | -1.250 | 1.954 | 0.523 | -5.112 | 2.612 |
|  |  | 20-29 | -0.300 | 2.048 | 0.884 | -4.347 | 3.747 |
|  |  | 40-49 | -1.560 | 2.077 | 0.454 | -5.664 | 2.544 |
|  |  | 50-59 | -0.473 | 2.286 | 0.836 | -4.991 | 4.046 |
|  | 40-49 | <20 | 0.310 | 0.971 | 0.750 | -1.610 | 2.230 |
|  |  | 20-29 | 1.260 | 1.148 | 0.274 | -1.009 | 3.529 |
|  |  | 30-39 | 1.560 | 2.077 | 0.454 | -2.544 | 5.664 |
|  |  | 50-59 | 1.087 | 1.534 | 0.479 | -1.944 | 4.118 |
|  | 50-59 | <20 | -0.777 | 1.363 | 0.569 | -3.471 | 1.917 |
|  |  | 20-29 | 0.173 | 1.494 | 0.908 | -2.780 | 3.126 |
|  |  | 30-39 | 0.473 | 2.286 | 0.836 | -4.046 | 4.991 |
|  |  | 40-49 | -1.087 | 1.534 | 0.479 | -4.118 | 1.944 |
| Package opening time | <20 | 20-29 | 2.650 | 1.397 | 0.060 | -0.111 | 5.411 |
|  |  | 30-39 | 2.750 | 3.008 | 0.362 | -3.196 | 8.696 |
|  |  | 40-49 | 2.030 | 1.495 | 0.177 | -0.925 | 4.985 |
|  |  | 50-59 | 0.786 | 2.099 | 0.708 | -3.361 | 4.934 |
|  | 20-29 | <20 | -2.650 | 1.397 | 0.060 | -5.411 | 0.111 |
|  |  | 30-39 | 0.100 | 3.152 | 0.975 | -6.130 | 6.330 |
|  |  | 40-49 | -0.620 | 1.767 | 0.726 | -4.113 | 2.873 |
|  |  | 50-59 | -1.864 | 2.300 | 0.419 | -6.410 | 2.683 |
|  | 30-39 | <20 | -2.750 | 3.008 | 0.362 | -8.696 | 3.196 |
|  |  | 20-29 | -0.100 | 3.152 | 0.975 | -6.330 | 6.130 |
|  |  | 40-49 | -0.720 | 3.197 | 0.822 | -7.039 | 5.599 |
|  |  | 50-59 | -1.964 | 3.520 | 0.578 | -8.920 | 4.993 |
|  | 40-49 | <20 | -2.030 | 1.495 | 0.177 | -4.985 | 0.925 |
|  |  | 20-29 | 0.620 | 1.767 | 0.726 | -2.873 | 4.113 |
|  |  | 30-39 | 0.720 | 3.197 | 0.822 | -5.599 | 7.039 |
|  |  | 50-59 | -1.244 | 2.361 | 0.599 | -5.910 | 3.423 |
|  | 50-59 | <20 | -0.786 | 2.099 | 0.708 | -4.934 | 3.361 |
|  |  | 20-29 | 1.864 | 2.300 | 0.419 | -2.683 | 6.410 |
|  |  | 30-39 | 1.964 | 3.520 | 0.578 | -4.993 | 8.920 |
|  |  | 40-49 | 1.244 | 2.361 | 0.599 | -3.423 | 5.910 |
| Donning time | <20 | 20-29 | 16.679 | 5.744 | 0.004* | 5.327 | 28.031 |
|  |  | 30-39 | 10.913 | 12.368 | 0.379 | -13.531 | 35.356 |
|  |  | 40-49 | 1.593 | 6.147 | 0.796 | -10.557 | 13.742 |
|  |  | 50-59 | -12.978 | 8.628 | 0.135 | -30.030 | 4.073 |
|  | 20-29 | <20 | -16.679 | 5.744 | 0.004* | -28.031 | -5.327 |
|  |  | 30-39 | -5.767 | 12.960 | 0.657 | -31.380 | 19.847 |
|  |  | 40-49 | -15.08667 | 7.266 | 0.040* | -29.446 | -0.728 |
|  |  | 50-59 | -29.658 | 9.457 | 0.002* | -48.348 | -10.967 |
|  | 30-39 | <20 | -10.913 | 12.368 | 0.379 | -35.356 | 13.531 |
|  |  | 20-29 | 5.767 | 12.960 | 0.657 | -19.847 | 31.380 |
|  |  | 40-49 | -9.320 | 13.144 | 0.479 | -35.297 | 16.657 |
|  |  | 50-59 | -23.891 | 14.471 | 0.101 | -52.490 | 4.709 |
|  | 40-49 | <20 | -1.593 | 6.147 | 0.796 | -13.742 | 10.557 |
|  |  | 20-29 | 15.087 | 7.266 | 0.040* | 0.728 | 29.446 |
|  |  | 30-39 | 9.320 | 13.144 | 0.479 | -16.657 | 35.297 |
|  |  | 50-59 | -14.571 | 9.707 | 0.136 | -33.756 | 4.614 |
|  | 50-59 | <20 | 12.978 | 8.628 | 0.135 | -4.073 | 30.030 |
|  |  | 20-29 | 29.658 | 9.457 | 0.002* | 10.967 | 48.348 |
|  |  | 30-39 | 23.891 | 14.471 | 0.101 | -4.709 | 52.490 |
|  |  | 40-49 | 14.571 | 9.707 | 0.136 | -4.614 | 33.756 |

Note: * p value is significant at 0.05.
