# Supplementary material for: Evaluating the influential factors for life preserver donning tests
Source: PLoS One. 2021 Feb 8;16(2):e0246705. doi: 10.1371/journal.pone.0246705 (PMC7870007; doi:10.1371/journal.pone.0246705)
Supplement: S2 Table — (DOCX) [file pone.0246705.s002.docx]

S2 Table. Pearson correlation between test subject characteristics and donning performance.

|  | Retrieving time | Package opening time | Donning time |
| --- | --- | --- | --- |
| Height | .152 (.062) | .224 (.006^*^) | .036 (.659) |
| Weight | .105 (.201) | .179 (.028^*^) | .035 (.670) |
| Head circumference | .055 (.500) | .170 (.037^*^) | .046 (.578) |

Note: The values outside and inside the parentheses are Pearson correlation coefficient and p value, respectively. * denotes that p < 0.05.
