# Supplementary material for: Evaluating the influential factors for life preserver donning tests
Source: PLoS One. 2021 Feb 8;16(2):e0246705. doi: 10.1371/journal.pone.0246705 (PMC7870007; doi:10.1371/journal.pone.0246705)
Supplement: S1 File — (DOCX) [file pone.0246705.s003.docx]

**The questionnaire and tool tests on the donning performance of traditional aviation life preservers**

PART1 ----------------------------------------------------------------------------------------------------------------------------

1.Name: .

2.Gender:Male□, Female □.

3.Age: .

4.Height: .

5.Domicile: .

Rural area □ , Urban area □.

**6. Fill in or measured by staff.**

Wearing glasses： Yes □. No □

Villager □ Student □

Head circumference: cm. Weight: cm. Body fat rate: .

BMI: . Metabolic rate: .

PART2 -------------------------------------------------------------------------------------------------------------------------------

According to your own situation, check mark in the corresponding strong, just strong, average, just weak, and weak.

Table 1. General intelligence

| Content | Strong,1 | Just strong,2 | Average, 3 | Just weak,4 | Weak,5 |
| --- | --- | --- | --- | --- | --- |
| 1. Learning new content quickly and easily |  |  |  |  |  |
| 2. Solving math problems quickly and correctly |  |  |  |  |  |
| 3. Academic performance |  |  |  |  |  |
| 4. Comprehension, analysis and comprehensive ability of texts |  |  |  |  |  |
| 5. Memory capacity of knowledge |  |  |  |  |  |

Table 2. Verbal ability

| Content | Strong,1 | Just strong,2 | Average, 3 | Just weak,4 | Weak,5 |
| --- | --- | --- | --- | --- | --- |
| 6. Ability to express one's own opinion |  |  |  |  |  |
| 7.Reading speed and comprehension |  |  |  |  |  |
| 8. Vocabulary |  |  |  |  |  |
| 9. Chinese performance |  |  |  |  |  |
| 10. Literary creation ability |  |  |  |  |  |

Table 3. Numerical aptitude

| Content | Strong,1 | Just strong,2 | Average, 3 | Just weak,4 | Weak,5 |
| --- | --- | --- | --- | --- | --- |
| 11.Accurate measurement capability |  |  |  |  |  |
| 12.Ability to calculate |  |  |  |  |  |
| 13. Mental arithmetic |  |  |  |  |  |
| 14. Abacus computing power |  |  |  |  |  |
| 15. Math scores |  |  |  |  |  |

Table 4. Spatial relation

| Content | Strong,1 | Just strong,2 | Average, 3 | Just weak,4 | Weak,5 |
| --- | --- | --- | --- | --- | --- |
| 16. Ability to solve spatial geometry |  |  |  |  |  |
| 17. Ability to draw three-dimensional graphics |  |  |  |  |  |
| 18. Three-dimensional sense of geometric figures |  |  |  |  |  |
| 19. Imaginative ability to expand the box into a floor plan |  |  |  |  |  |
| 20. Imagination of three-dimensional objects |  |  |  |  |  |

Table 5. Shape perception ability

| Content | Strong,1 | Just strong,2 | Average, 3 | Just weak,4 | Weak,5 |
| --- | --- | --- | --- | --- | --- |
| 21.Ability to discover nuances of similar graphics |  |  |  |  |  |
| 22. Recognition ability of object details |  |  |  |  |  |
| 23. Attention to object details |  |  |  |  |  |
| 24. Ability to observe whether the object image is correct |  |  |  |  |  |
| 25. Ability to describe objects in detail |  |  |  |  |  |

Table 6. Clerical awareness

| Content | Strong,1 | Just strong,2 | Average, 3 | Just weak,4 | Weak,5 |
| --- | --- | --- | --- | --- | --- |
| 26.Ability to copy information quickly and accurately |  |  |  |  |  |
| 27. Ability to spot typos |  |  |  |  |  |
| 28. Ability to detect calculation errors |  |  |  |  |  |
| 29. Search ability of coded cards |  |  |  |  |  |
| 30. Self-control |  |  |  |  |  |

Table 7. Motor coordination ability

| Content | Strong,1 | Just strong,2 | Average, 3 | Just weak,4 | Weak,5 |
| --- | --- | --- | --- | --- | --- |
| 31. Playing electronic games |  |  |  |  |  |
| 32. Activities such as playing basketball, volleyball, and football |  |  |  |  |  |
| 33. Playing table tennis and badminton |  |  |  |  |  |
| 34. Ability to use an abacus |  |  |  |  |  |
| 35. Typing ability |  |  |  |  |  |

Table 8.Finger flexibility

| Content | Strong,1 | Just strong,2 | Average, 3 | Just weak,4 | Weak,5 |
| --- | --- | --- | --- | --- | --- |
| 36. Using small tools dexterously |  |  |  |  |  |
| 37. Activities that use fingers such as piercing and knitting |  |  |  |  |  |
| 38.Using your fingers to make a small craft |  |  |  |  |  |
| 39.Dexterity of using the counter |  |  |  |  |  |
| 40. Play the piano |  |  |  |  |  |

Table 9.Wrist flexibility

| Content | Strong,1 | Just strong,2 | Average, 3 | Just weak,4 | Weak,5 |
| --- | --- | --- | --- | --- | --- |
| 41.Sorting things by hand |  |  |  |  |  |
| 42. Hand flexibility when pushing and pulling things |  |  |  |  |  |
| 43. Peeling the apple quickly |  |  |  |  |  |
| 44. Flexibility to use hand tools |  |  |  |  |  |
| 45. Flexibility in manual activities such as painting and carving |  |  |  |  |  |

PART3 ----------------------------------------------------------------------------------------------------------------------------

Tool tests included placing tool test, turning tool test, assembling tool test, and disassembling tool test. These tests required the test subjects can complete placing task, turning task, assembling task, and disassembling task as quickly as possible. The results of the experiment were recorded by the staff.

Table 10. Tool tests

| Content | Time |
| --- | --- |
| 1.Placing |  |
| 2.Turning |  |
| 3.Assembling |  |
| 4.Disassembling |  |
