# Supplementary material for: Evaluating the influential factors for life preserver donning tests
Source: PLoS One. 2021 Feb 8;16(2):e0246705. doi: 10.1371/journal.pone.0246705 (PMC7870007; doi:10.1371/journal.pone.0246705)
Supplement: Attachment — Submitted filename: Review.docx [file pone.0246705.s005.docx]

It is a messy article without suitable coherence.

The subject characteristics must be presented in the materials and method section.

All tables must be presented at result section.

Some tables, such as Table 7, are too long and is not suitable by this way for an article.

The discussion section should be rewritten based on the essay writing framework.

The article needs a general review.
